# Supplementary material for: Impact of reduction of susceptibility to SARS-CoV-2 on epidemic dynamics in four early-seeded metropolitan regions
Source: Sci Rep. 2021 Jun 9;11:12213. doi: 10.1038/s41598-021-91247-7 (PMC8190298; doi:10.1038/s41598-021-91247-7)
Supplement: Supplementary file 1 — Supplementary Information. [file 41598_2021_91247_MOESM1_ESM.pdf]

Impact of reduction of susceptibility to SARS-CoV-2 on epidemic dynamics in four early-seeded metropolitan regions (Supplementary Material)

Thomas J. Barrett[1], Karen C. Patterson[2,3], Timothy M. James[1], and Peter Krüger[1,*]

[1]School of Mathematical and Physical Sciences, University of Sussex, Brighton BN1 9QH, UK
[2]Brighton and Sussex Medical School, University of Sussex, Brighton BN1 9PX, UK
[3]Host-Pathogen Interactions in Tuberculosis Laboratory, The Francis Crick Institute, London NW1 1AT, UK
[*]P.Kruger@sussex.ac.uk

Exponential Growth / Decay Time Constants

**Area**	**Initial Growth** $1/D_u$ (days)	**Decline** $1/D_l$ (days)
UK	3.29 ± 0.93	-
Rest of England	4.22 ± 0.83	-22.0 ± 1.0
London	3.49 ± 0.66	-14.1 ± 1.2
Spain	3.0 ± 1.1	-
Rest of Spain	2.52 ± 0.34	-23.2 ± 3.8
Madrid	2.58 ± 0.97	-18.0 ± 1.7
Italy	3.43 ± 0.77	-
Rest of Italy	3.83 ± 0.52	-33.6 ± 3.7
Lombardy	3.5 ± 1.7	-24.3 ± 3.7
US	3.72 ± 0.50	-
Rest of US	4.07 ± 0.95	-56 ± 27
New York	2.72 ± 0.37	-15.7 ± 0.9

Table S1. Exponential growth/decline time constants. Linear fits to the logarithm of daily deaths per million population data are used to avoid heteroskedastic bias. Fits to the initial growth phase for the total country level data in Fig. 1a of the main paper produce time constant values which are all consistent with each other, with a mean of $\bar{\tau} = (3.4 \pm 0.2)$ days. Country level data are then broken down into main metropolitan region and rest of country (Fig. 2 upper panels of main paper), with corresponding fits to both the exponential growth and decline phases listed. Errors are 2 standard deviation confidence intervals (CI) from fits.

Behavioural Data Examples for Additional Regions

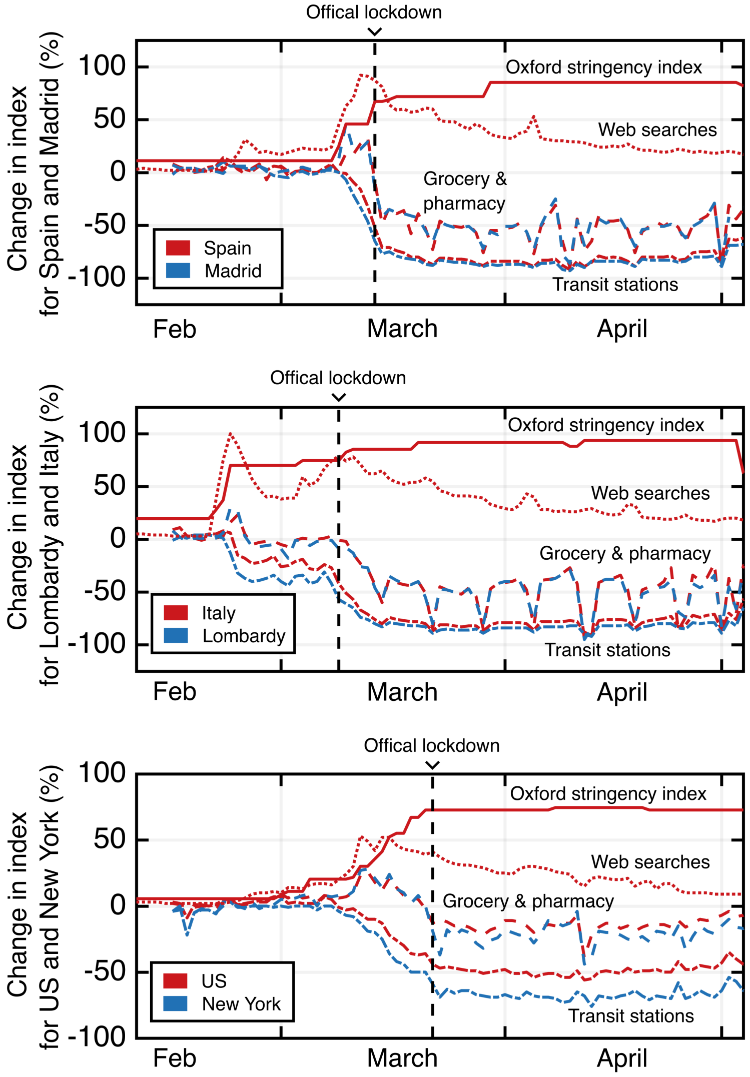

Figure S1. Comparison of different metrics that potentially affect infection contact rates and their time-dependence during the first introduction of public health interventions in early-2020, shown for Spain/Madrid (top), Italy/Lombardy (middle), and United States/New York (bottom). Google location data are shown for the *Transit Stations* and *Grocery & Pharmacy* categories for the whole of each country and the main metropolitan region, together with both the national *Oxford Stringency Index* and change in frequency of web searches for the keyword "coronavirus" for each. The vertical dashed lines indicate the official national lockdown dates, except in the United States where restrictions were introduced progressively state by state. In this case, the date of the stay-at-home order for the example of New York is shown instead.
